# Editorial for Special Issue “Leaf Senescence” in *Plants*

**DOI:** 10.3390/plants10081490

**Published:** 2021-07-21

**Authors:** Ulrike Zentgraf, Ana G. Andrade, Jasmin Doll

**Affiliations:** Centre for Molecular Biology of Plants, University of Tuebingen, Auf der Morgenstelle 32, 72076 Tuebingen, Germany; ana.andrade@zmbp.uni-tuebingen.de (A.G.A.); jasmin.doll@zmbp.uni-tuebingen.de (J.D.)

Senescence in plants is often described as the last step in the life history of a plant. However, senescence takes place from early on throughout development and a plant can sacrifice older leaves for the sake of the whole plant. Leaf senescence aims at remobilizing previously acquired nitrogen, carbon and mineral resources out of the senescing tissue into developing parts of the plant, before the leaf eventually dies and is shed. Before anthesis, sequential leaf senescence leads to the repartitioning of nutrients from older leaves to newly developing non-reproductive organs. After anthesis, monocarpic leaf senescence governs nutrient reallocation to the now developing reproductive organs and, therefore, has a very critical impact on yield. During monocarpic senescence, potentially all the leaves of a plant can undergo senescence, leading to the death of the whole plant (Figure 1). 

In the last two decades, it became obvious that no “master regulator” for senescence exists but an extremely complex regulatory network controls all aspects of senescence. Leaf and plant age are the predominant parameters controlling the onset and the progression of developmental senescence; however, we still do not understand exactly how these parameters are sensed by the plants. Moreover, incoming environmental signals are constantly integrated and long-lasting unfavorable conditions have the potential to induce senescence prematurely. Premature senescence serves as an exit-strategy to produce offspring when biotic or abiotic stresses are encountered over longer periods and produce severe damages. As a tradeoff, seed quantity and quality are often diminished and can significantly lower the productivity of plants. In crop plants, stress-induced premature senescence can have a major impact on yield, referring to quantity and quality. Moreover, post-harvest senescence can change nutrient contents and the profile of volatile organic compounds, which relate, for example, to the aroma of vegetables or salads. As well in this case, stress conditions during harvest, storage and transportation can influence the onset and progression of post-harvest senescence with a high impact on shelf life performance [1].

Transcriptional regulation was discovered to be one of the main drivers of senescence processes, implying a central role for transcription factors of many different transcription factor families in model plants as well as in crop plants [2,3,4]. However, in most cases, no simple signal transduction pathways are realized, but complex multi-layer regulatory cues including many feedback loops are in place to control senescence. Even for a single transcription factor gene, transcriptional, post-transcriptional, as well as post-translational regulatory mechanisms can act in concert [4]. In addition, the turn-over of specific regulators can also be tightly controlled with the degradation of damaged proteins by the 20S proteasome playing an important role [5]. Moreover, we are just beginning to understand the dynamic changes in chromatin structure and nuclear architecture during senescence. Alternative splicing and polyadenylation events have rarely been analyzed. 

As signaling components, almost all plant hormones are involved in senescence regulation, which also pinpoints the high cross regulation to many other processes [6]. There is a large overlap and crosstalk between senescence-related processes and pathogen responses [7]. Reactive oxygen species have also been characterized as participating, especially in the early signaling events of leaf senescence and acting in concert with salicylic acid. In addition, less well-known signaling molecules such as melatonin are engaged in the control of the scavenging systems for reactive oxygen species in grape leaves and, by that, influence the expression of senescence-associated genes [8]. On this note, the role of chloroplasts was revisited in the recent years and it became clear that the redox balance in chloroplasts significantly contributes to senescence timing and progression [9].

Essential for the efficient remobilization of carbon- and nitrogen-containing compounds is not only the systematic degradation of macromolecules but also the conversion of their degradation products into transport forms and the activation of transport processes per se. Specific amino acids such as aspartate are enriched during the onset of leaf senescence and are part of the leaf curing process in tobacco leaves. For this purpose, specific enzymes have to be activated to increase the levels of specific amino acids [10]. Moreover, the expression of specific members of transporter families are activated during senescence to guarantee the increasing demand and correct the directionality of the transport of sugars and amino acids out of the senescing tissue to pods and seeds [11]. 

This Special Issue combines a wide range of senescence topics in five articles and six reviews on plant senescence processes. Work from all over the world including Europe (UK, Germany, Switzerland), Asia (China, Pakistan), South America (Argentina) and New Zealand was brought together, indicating that this is an important and “hot” topic in all parts of the world. Changing climate conditions will most likely produce long lasting unfavorable conditions and will lead to premature senescence induction and yield losses in many crop plants. Therefore, understanding senescence processes, their regulation and the impact of stress conditions in model and crop plants will be one of our future challenges. Increasing knowledge in this field will hopefully contribute to new transgenic lines and breeding strategies for plants with improved stress tolerance and optimized senescence programs.

## Figures and Tables

**Figure 1:**
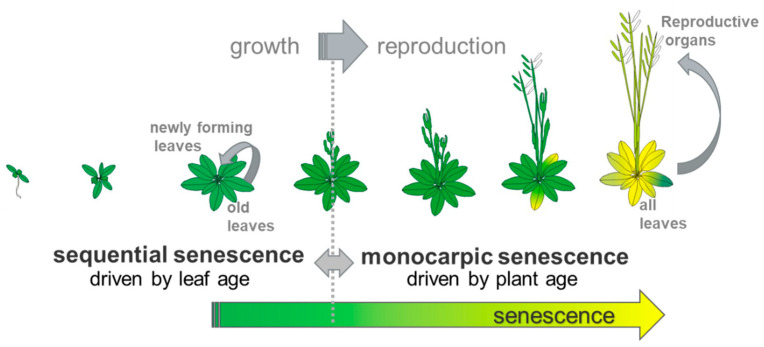
Leaf senescence in the model plant *Arabidopsis thaliana*. Leaf senescence takes place all over development. At the transition from the growth to reproduction phase, senescence is switched from sequential to monocarpic senescence.
